# The Role of α-Enolase on the Production of Interleukin (IL)-32 in Con A-Mediated Inflammation and Rheumatoid Arthritis (RA)

**DOI:** 10.3390/ph17040531

**Published:** 2024-04-20

**Authors:** Hyejung Jo, Seulgi Shin, Tomoyo Agura, Seoyoun Jeong, Hyovin Ahn, Junmyung Lee, Yejin Kim, Jae Seung Kang

**Affiliations:** 1 Laboratory of Vitamin C and Antioxidant Immunology, Department of Anatomy and Cell Biology, Seoul National University College of Medicine, Seoul 03080, Republic of Korea; luv_jo@snu.ac.kr (H.J.); tomoyoagura@snu.ac.kr (T.A.); jsy9804@snu.ac.kr (S.J.); jahb1220@snu.ac.kr (H.A.); leejm@snu.ac.kr (J.L.); bbambaya921@snu.ac.kr (Y.K.); 2 Institute of Allergy and Clinical Immunology, Seoul National University Medical Research Center, Seoul 03080, Republic of Korea; seulgi.shin@snu.ac.kr; 3 Artificial Intelligence Institute, Seoul National University, Seoul 08826, Republic of Korea; 4 Department of Applied Bioengineering, Graduate School of Convergence Science and Technology, Seoul National University, Seoul 08826, Republic of Korea

**Keywords:** inflammation, ENO1, IL-32, RA

## Abstract

Interleukin (IL)-32 is produced by T lymphocytes, natural killer cells, monocytes, and epithelial cells. IL-32 induces the production of pro-inflammatory cytokines such as tumor necrosis factor (TNF)-α, IL-1β, IL-6, and IL-8, and IL-32 expression is highly increased in rheumatoid arthritis (RA) patients. Enolase-1 (ENO1) is a glycolytic enzyme and the stimulation of ENO1 induces high levels of pro-inflammatory cytokines in concanavalin A (Con A)-activated peripheral blood mononuclear cells (PBMCs) and macrophages in RA patients. In addition, there are many reports that anti-ENO1 antibody is correlated with the disease progression of RA. It implies that ENO1 could regulate IL-32 production during inflammation related to the pathogenesis of RA. Therefore, we investigated the role of ENO1 in IL-32 production using Con A-activated PBMCs and RA PBMCs. IL-32 expression is increased by ENO1 stimulation using real-time PCR and ELISA. In addition, we confirmed that IL-32 production was decreased in Con A-activated PBMCs and RA PBMCs pre-treated with NF-κB or p38 MAPK pathway inhibitors. Taken together, these results suggest that ENO1 plays an important role in inflammation through the induction of IL-32 production by the activation of the NF-κB and p38 MAPK pathways.

## 1. Introduction

Interleukin (IL)-32 is considered a pro-inflammatory cytokine and is produced by T lymphocytes, natural killer (NK) cells, monocytes, and epithelial cells. The genomic structure of IL-32 is organized into eight exons and located on human chromosome 16p13.3, and IL-32 exists in nine isoforms; IL-32α, IL-32β, IL-32γ, IL-32δ, IL-32θ, IL-32ε, IL-32ζ, IL-32η, and IL-32s. IL-32γ is the full-length isoform without any exonic deletions and the most active isoform of the cytokine [1,2,3]. IL-32 induces pro-inflammatory cytokines such as tumor necrosis factor (TNF)-α, IL-1, IL-6, and IL-8 through the activation of nuclear factor (NF)-κB and p38 mitogen-activated protein kinase (MAPK) [4,5,6]. IL-32 is known to play an important role in inflammatory diseases such as inflammatory bowel diseases (IBD) and rheumatoid arthritis (RA). The expression level of IL-32 in the synovium of patients with RA is highly increased. In addition, IL-32 injection into the knee joints of mice resulted in joint swelling, infiltration of immune cells, and cartilage damage [1,3,5].

Enolase (ENO) is a glycolytic enzyme that degrades 2-phosphoglycerate to 2-phosphoenolpyruvate in the last steps of the catabolic glycolytic pathway. The enzyme occurs as three isoforms: α-enolase (enolase-1 [ENO1]) is expressed in most tissues, whereas β-enolase (enolase-3 [ENO3]) is predominantly found in muscle tissues, and γ-enolase (enolase-2 [ENO2]) is found in neuron and neuroendocrine tissues [7,8]. ENO1 is mainly expressed in the cytosol. However, ENO1 is detected on the surface of hematopoietic cells; monocytes, T cells, B cells, neuronal cells, and endothelial cells during pathological conditions such as inflammation, autoimmunity, and malignancy [9,10,11]. ENO1 expression is increased on the cell surface by lipopolysaccharide (LPS), phorbol myristate acetate (PMA), and concanavalin (Con) A stimulation and it is involved in many functions for inflammatory responses [11,12]. There are many reports that ENO1 antibodies play pathogenic roles in a variety of autoimmune and inflammatory diseases such as systemic lupus erythematosus, systemic sclerosis, Behcet’s disease, ulcerative colitis, Crohn’s disease, retinopathy, and RA [7,13,14,15]. RA is a systemic autoimmune inflammatory disease that affects mostly multiple peripheral joints [16] and includes synovial inflammation, pannus formation, and subsequent bone destruction [17,18,19]. Although the exact mechanisms that contribute to the pathogenesis are still unknown, it is well accepted that many cells such as T cells, B cells, fibroblast-like synoviocytes, antigen-presenting cells, and their extensive production of pro-inflammatory mediators such as TNF-α, IL-1, IL-6, IL-15, IL-17, and IL-18 are involved [20,21].

In the previous study, Con A stimulation increased the expression of ENO1 on the surface of peripheral blood mononuclear cells (PBMCs), resulting in increased production of pro-inflammatory cytokines through the NF-κB and p38 MAPK pathways [11]. Although there are several reports regarding the role of IL-32 in the pathogenesis of RA, it is not yet clear whether ENO1 is involved in IL-32 production. Therefore, we investigated the role of ENO1 in the production of IL-32 under inflammatory conditions using Con A-activated PBMCs and RA PBMCs through the activation of the NF-κB and p38 MAPK pathways.

## 2. Results

### 2.1. IL-32 Is Induced by ENO1 Stimulation in Con A-Activated PBMCs

**Figure 1:**
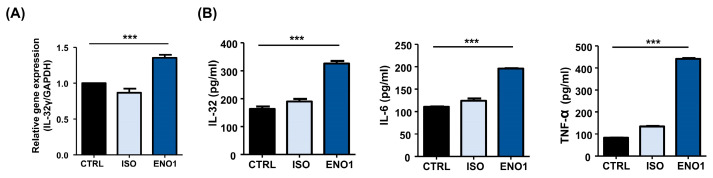
IL-32 mRNA expression by ENO1 stimulation from Con A-activated PBMCs. Isolated PBMCs from healthy individuals were stimulated with Con A for 24 h. (**A**) Activated PBMCs were collected at 6 h after stimulation with anti-ENO1 mAb for 1 h. MOPC21 was used as an isotype control. Real-time PCR was performed by using the specific probe for IL-32γ as described in Section 4. (**B**) Activated PBMCs were stimulated with anti-ENO1 mAb and incubated for 48 h. MOPC21 was used as an isotype control. The supernatant was harvested and the production of IL-32, IL-6, and TNF-α was determined by ELISA. Data were collected from three independent experiments and presented as the means ± SD. *** *p* < 0.001.

To investigate whether IL-32 expression is increased in Con A-activated PBMCs by ENO1 stimulation, PBMCs were obtained from healthy individuals and activated with Con A for 48 h. After activation, the PBMCs were stimulated with anti-ENO1 mAb. As shown in Figure 1A, B, IL-32γ mRNA expression and IL-32 protein levels were increased by ENO1 stimulation compared with the control without stimulation. Based on the report, ENO1 stimulation increases the production of pro-inflammatory cytokines, including IL-1α/β, IL-6, IL-18, and TNF-α, from RA PBMCs [11]. We also confirmed that pro-inflammatory IL-6 and TNF-α were increased by stimulation with ENO1 using ELISA (Figure 1B). This result indicates that IL-32 expression is upregulated by ENO1 stimulation from Con A-activated PBMCs.

### 2.2. IL-32 Production Is Increased in Con A-Activated PBMCs by ENO1 Stimulation through the Activation of NF-κB and p38 MAPK

**Figure 2:**
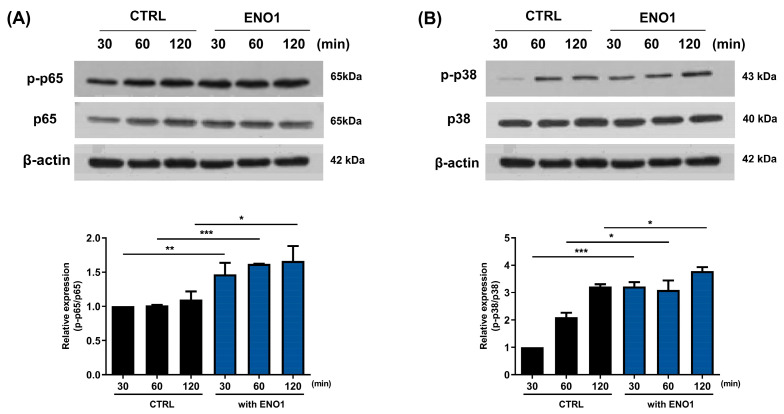
Increase in the phosphorylation of NF-κB and p38 MAPK in Con A-activated PBMCs by ENO1 stimulation. PBMCs were incubated with Con A for 48 h and stimulated with anti-ENO1 mAb for 30, 60, and 120 min. Cells were lysed and protein was extracted for Western blot analysis as described in Section 4. The expression of (**A**) p-p65 and (**B**) p-p38 were examined using Western blot analysis. Data were represented as relative intensity of phosphorylated form to total form, p-p65/p65 and p-p38/p38, and normalized by the control intensity as 1. These results are representative of three independent experiments. Data are presented as means ± SD. * *p* < 0.05, ** *p* < 0.01, *** *p* < 0.001.

It has been reported that ENO1 increases the production of pro-inflammatory mediators through the activation of the NF-κB and p38 MAPK pathways in Con A-activated PBMCs and RA PBMCs [11]. We confirmed that the NF-κB and p38 MAPK pathways were activated in a time-dependent manner by ENO1 stimulation using Western blot analysis. The phosphorylation of p65 was increased and peaked at 120 min, and the phosphorylation of p38 MAPK increased at 30 min after stimulation (Figure 2).

**Figure 3:**
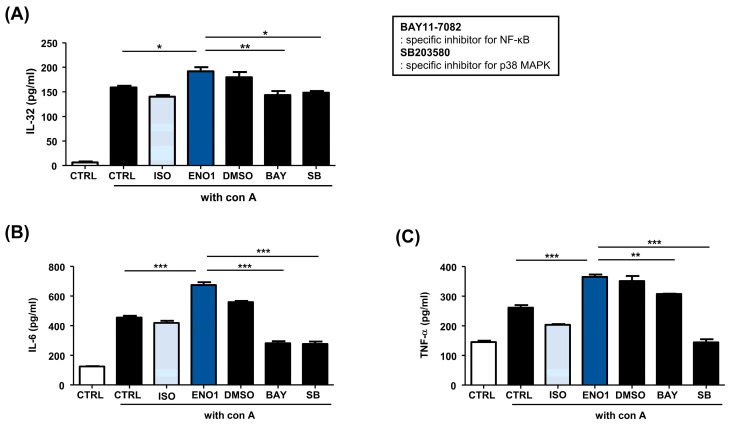
Inhibition of IL-32 production by the pre-treatment of pathway inhibitors in Con A-activated PBMCs. After Con A activation, PBMCs were pre-treated with DMSO (vehicle control), BAY11-7082 (2.5 μM), or SB203580 (40 μM) for 1 h and then stimulated with anti-ENO1 mAb for 1 h. MOPC21 was used as an isotype control. After incubation for 48 h, culture media were collected and centrifuged at 600× *g* for 10 min. The supernatants were harvested and (**A**) IL-32, (**B**) IL-6, and (**C**) TNF-α amounts were examined using ELISA. These results are representative of three independent experiments. Data are presented as means ± SD. * *p* < 0.05, ** *p* < 0.01, *** *p* < 0.001.

Subsequently, we examined whether the activation of NF-κB and p38 MAPK are involved in ENO1-induced IL-32 production in Con A-activated PBMCs. Con A-activated PBMCs were pre-treated with BAY11-7082 (an NF-κB inhibitor) and SB203580 (an p38 MAPK inhibitor) for 1 h and then stimulated with anti-ENO1 mAb. Although ENO1 stimulated PBMCs, ENO1-induced IL-32 production and pro-inflammatory cytokines were decreased in PBMCs treated with inhibitors (Figure 3). Taken together, we suggest that ENO1 increases IL-32 production via the activation of NF-κB and p38 MAPK.

### 2.3. IL-32 Is Induced by ENO1 Stimulation in RA PBMCs

**Figure 4:**
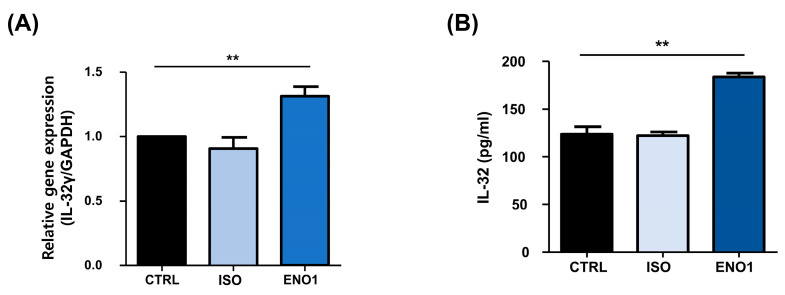
ENO1-induced IL-32 mRNA expression and production in RA PBMCs. PBMCs separated from RA patients were stimulated with anti-ENO1 mAb or MOPC21 (isotype control) for 1 h. (**A**) After incubation for another 6 h, total RNA was extracted and cDNA was made. Real-time PCR was performed using the specific primer for IL-32γ as described in Section 4. Results were expressed as relative intensity and each group was adjusted to that of β-actin. (**B**) Isolated RA PBMCs were stimulated with anti-ENO1 mAb and incubated for 48 h. The cultured supernatant was collected and the production of IL-32 was measured by ELISA. Data are presented as the means ± SD. ** *p* < 0.01.

Con A has been used to stimulate the PBMCs to establish a model of inflammatory diseases, including rheumatoid arthritis, which is a systemic autoimmune disease characterized by synovial inflammation and the destruction of bone and cartilage [11,17,18]. As shown in Figure 1, IL-32 mRNA expression and its protein production were increased by ENO1 stimulation from Con A-activated PBMCs. To confirm this result, we examined the role of ENO1 in IL-32 production using RA PBMCs. After PBMCs were obtained from RA patients and stimulated with anti-ENO1 mAb, we measured the expression of IL-32 at the mRNA and its protein production using real-time PCR and ELISA. IL-32γ mRNA expression and IL-32 production were increased compared to the control (Figure 4). These results indicate that IL-32 mRNA expression and production are increased in RA PBMCs by ENO1 stimulation.

### 2.4. IL-32 Production Is Increased in RA PBMCs by ENO1 Stimulation through the Activation of NF-κB and p38 MAPK

**Figure 5:**
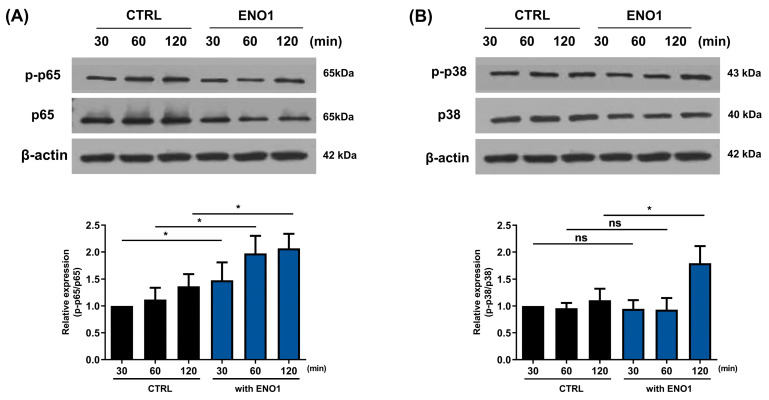
Increase in the phosphorylation of NF-κB and p38 MAPK in RA PBMCs by ENO1 stimulation. RA PBMCs were stimulated with anti-ENO1 mAb for 30, 60, and 120 min. Cells were lysed and protein was extracted for Western blot analysis as described in Section 4. The expressions of (**A**) p-p65 and (**B**) p-p38 were examined using Western blot analysis. Data were represented as relative intensity of phosphorylated form to total form, p-p65/p65 and p-p38/p38, and normalized by the control intensity as 1. These results are representative of three independent experiments. Data are presented as means ± SD. * *p* < 0.05; ns: not significant.

**Figure 6:**
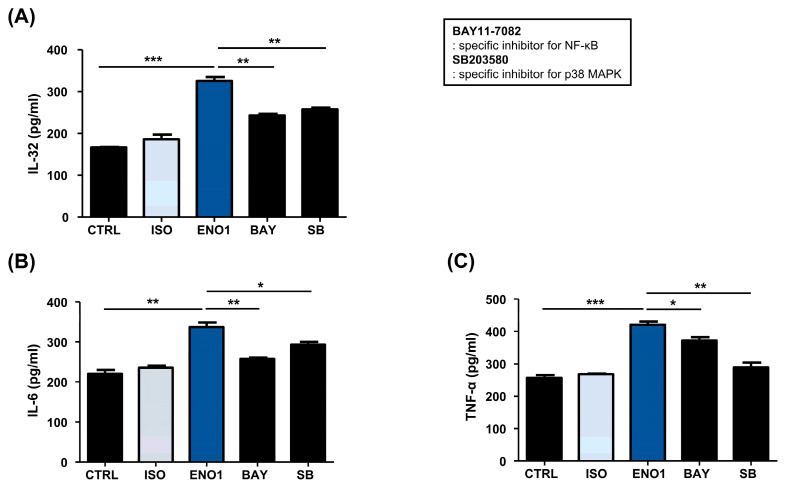
Inhibition of IL-32 production by the pre-treatment of pathway inhibitors in RA PBMCs. After Con A activation, PBMCs were pre-treated with BAY11-7082 (2.5 μM) and SB203580 (40 μM) for 1 h and then stimulated with anti-ENO1 mAb for 1 h. MOPC21 was used as an isotype control. After incubation for 48 h, culture media were collected and centrifuged at 600× *g* for 10 min. The supernatants were harvested and (**A**) IL-32, (**B**) IL-6, and (**C**) TNF-α amounts were examined using ELISA. These results are representative of three independent experiments. Data are presented as means ± SD. * *p* < 0.05, ** *p* < 0.01, *** *p* < 0.001.

To investigate whether the activation of NF-κB and p38 MAPK is involved in ENO1-induced IL-32 production in RA PBMCs, we performed Western blot analysis. As shown in Figure 5, phosphorylation of p65 and p38 MAPK was increased in the PBMCs from RA patients after ENO1 stimulation compared to the control. Subsequently, the RA PBMCs were pre-treated with NF-κB and p38 MAPK inhibitor and then stimulated with anti-ENO1 mAb. We found that ENO1-induced IL-32 production and pro-inflammatory cytokines were inhibited (Figure 6). These results suggest that ENO1 increases the production of IL-32 in RA PBMCs through NF-κB and p38 MAPK.

## 3. Discussion

A previous study demonstrated that ENO1 expressed on the surface of monocytes and macrophages contributes to the production of pro-inflammatory mediators in rheumatoid arthritis [11]. Several studies reported that ENO1 and IL-32 play pathogenic roles in a variety of inflammatory diseases [4,6,14,15]. However, it is not yet clarified whether ENO1 is involved in the production of IL-32 under inflammatory conditions. Previous reports, highlighting the role of IL-32 in the pathogenesis of inflammatory diseases, suggest that ENO1 might be an important stimulator in the production of IL-32. Therefore, in this study, we primarily investigated the role of ENO1 in IL-32 synthesis in Con A-mediated PBMCs and RA PBMCs derived from patients with rheumatoid arthritis. Our findings revealed that Abs targeting ENO1 induced the production of IL-32 and pro-inflammatory mediators such as IL-6 and TNF-α. We show increased IL-32 expressions at transcription and production in both Con A-activated PBMCs and PBMCs obtained from RA patients by ENO1 stimulation (Figure 1 and Figure 4). As shown in Figure 2 and Figure 5, ENO1 stimulation significantly increases the activation of NF-κB and p38 MAPK, and the production of IL-32 and pro-inflammatory cytokines was decreased after treatment with NF-κB or p38 MAPK inhibitors (Figure 3 and Figure 6). This finding means that NF-κB and p38 MAPK play a crucial role in the production of mediators, especially IL-32, during the inflammatory process induced by ENO1.

As previously mentioned, IL-32 stimulates the production of various inflammatory mediators through the NF-κB and p38 MAPK pathways and has an important role in the progression of various inflammatory disorders such as RA and IBD [22,23]. In this study, to investigate the signaling pathways involved in RA, we used BAY11-7082 as a specific NF-κB inhibitor and SB203580 as a p38 MAPK inhibitor. There may be some possible limitations in this study. First, only one NF-κB pathway inhibitor was used. BAY11-7082 specifically inhibits the phosphorylation and degradation of IκBα and prevents NF-κB from entering the nucleus, followed by an inhibition of the transcription of target genes [24,25]. For this reason, BAY11-7082 is a commonly used inhibitor of the NF-κB pathway and many studies used BAY11-7082 as a specific inhibitor of NF-κB [26,27]. However, BAY11-7082 is also known as a broad-spectrum inhibitor [28]. In addition, there are various p38 MAPK inhibitors, such as FR167653, SB239063, and SB202190, and SB203580, which inhibits p38 MAPK catalytic activity by binding to the ATP binding pocket, different from the mechanisms of the other inhibitors [29]. Therefore, the results of this study need to be further verified using other inhibitors in the future.

ENO1 and IL-32 commonly act on the development and progression of cancer [9,12,30,31,32]. Considering that inflammation is one of the critical factors for the initiation of tumor development and progression, IL-32 production by ENO1 stimulation is closely related to tumorigenesis. Based on the report, IL-32 is highly expressed in patients with pancreatic cancer or gastric cancer and IL-32 has pro-cancer effects that inhibit apoptosis, stimulate DNA synthesis in the proliferation of cancer cells, and increase invasion associated with tumor progression and metastasis [33,34,35,36]. Likewise, ENO1 is expressed in pancreatic cancer and liver cancer cells and ENO1 promotes cell proliferation, migration, invasion, and tumorigenesis in non-small cell lung cancer [37,38,39,40,41]. Therefore, we expect that IL-32 regulation by ENO1 could be applied to treat cancers with inflammatory responses as well as inflammatory diseases.

## 4. Materials and Methods

### 4.1. Isolation of PBMCs

Heparinized peripheral blood was collected from healthy volunteers and RA patients. Peripheral blood was mixed with an equal volume of phosphate-buffered saline (PBS) and peripheral blood mononuclear cells (PBMCs) were isolated with a density gradient centrifuge using Ficoll-Paque™ PLUS (GE Healthcare Biosciences, Uppsala, Sweden). After centrifugation at 2000 rpm for 20 min, a buffy coat was collected and washed twice with PBS. The residual red blood cells were lysed by red blood cell lysis buffer (Sigma-Aldrich, St. Louis, MO, USA) for 5 min. After washing cells twice with PBS, cells were cultured in RPMI 1640 (WELGENE, Daegu, Republic of Korea) supplemented with 10% heat-inactivated fetal bovine serum (WELGENE), 100 U/mL of penicillin, and 100 μg/mL streptomycin (WELGENE) at 37 °C in a humidified incubator containing 5% CO_2_.

### 4.2. Stimulation of PBMCs with Concanavalin A (Con A) and α-Enolase (ENO1)

PBMCs from healthy volunteers were stimulated with 2 μg/mL Con A (Calbiochem, Darmstadt, Germany) for 48 h in a 37 °C incubator. After Con A stimulation, PBMCs were washed twice with PBS. Con A-activated PBMCs and PBMCs from RA patients were stimulated with anti-ENO1 mAb (1 μg/10^6^ cells) at room temperature with rotation for 1 h. MOPC-21 (1 μg/10^6^ cells; Sigma-Aldrich) was used as an isotype control antibody. After ENO1 stimulation, cells were transferred to a 24-well plate and incubated in a 37 °C incubator.

### 4.3. Real-Time Polymerase Chain Reaction (Real-Time PCR)

To study the expression of IL-32 in Con A-activated PBMCs and PBMCs from RA patients, real-time PCR was performed. Cells (2 × 10^6^) were harvested at 1, 2, 4, and 6 h after ENO1 stimulation. Total RNA was extracted with TRIzol (Invitrogen, Carlsbad, CA, USA) and complementary DNA was synthesized using 1 µg of total RNA with the Reverse Transcription System (Promega, Madison, WI, USA). The results were performed using the Rotor-Gene SYBR Green PCR kit (Qiagen, Hilden, Germany) and Rotor-Gene Q 2plex-real-time RT PCR instrument (Qiagen). Amplification was performed using the SYBR Green master mix (Qiagen). The primer used for the real-time PCR was as follows: 5′-GTAATGCTCCTCCCTACTTC-3′ (forward) and 5′-GCAAAGGTGGTGTCAGTATC-3’ (reverse) for IL-32γ; 5′-GTGGGGCGCCCCAGGCACCA-3′ (forward) and 5′-CTCCTTAATGTCACG CACGATTTC-3’ (reverse) for β-actin.

### 4.4. Enzyme-Linked Immunosorbent Assay (ELISA)

After Con A activation of PBMCs, cells (5 × 10^6^) were stimulated with anti-ENO1 mAb (1 μg/10^6^ cells) at room temperature with rotation for 1 h and incubated for 48 h. The concentration of IL-32, TNF-α, and IL-6 in cultured supernatant was measured using an ELISA kit (R&D systems, Minneapolis, MN: IL-32 and TNF-α; Biolegend, San Diego, CA, USA: IL-6) according to the manufacturer’s instruction. The relative absorbance was measured at 450 nm using the SoftmaxPro software v.7.1 (Molecular Devices, Sunnyvale, CA, USA).

### 4.5. Inhibitor Study for Signal Pathway

Specific inhibitors of NF-κB (Bay 11-7082) and p38 MAPK (SB203580) were purchased from Sigma-Aldrich. These inhibitors were used to identify the signaling pathways involved in the induction of cytokines by ENO1 stimulation with anti-ENO1 mAb. Con A-activated PBMCs and PBMCs from RA patients (5 × 10^6^) were pre-treated with DMSO (vehicle control) or inhibitors (Bay 11-7082: 2.5 μM, SB203580: 40 μM) for 1 h and washed twice with PBS. After washing, cells were stimulated with anti-ENO1 mAb (1 μg/10^6^ cells) at room temperature with rotation for 1 h.

### 4.6. Western Blot Analysis

After ENO1 stimulation with anti-ENO1 mAb, cells (2 × 10^6^) were lysed, and proteins were extracted in a lysis buffer containing 50 mM Tris-HCl (pH 7.4), 1% NP-40, 0.25% sodium deoxycholate, 150 mM NaCl, 1 mM EDTA, and protease inhibitor and phosphatase inhibitor cocktails (Sigma-Aldrich). An equal amount of protein at 30 μg per sample was dissolved in a 10% polyacrylamide–SDS gel with 100 V for 4 h and transferred onto a nitrocellulose membrane. Blocking was performed at room temperature for 1 h with 5% nonfat milk in PBS containing 0.05% Tween 20 (PBS-T). The blocked membrane was incubated with anti-p65 Ab (1:200; Santa Cruz Biotechnology, Dallas, TX, USA), anti-phospho-p65 Ab (1:500; Cell signaling Technology, Boston, MA, USA), anti-p38 MAPK Ab, anti-phospho-p38 MAPK Ab, and anti-β-actin Ab (1:1000; Cell signaling Technology) at 4 °C overnight. After washing thrice with PBS-T, the membrane was incubated with horse radish peroxidase (HRP)-conjugated anti-rabbit IgG secondary Ab (1:5000; Cell signaling Technology) for anti-p65, anti-phospho-p65, anti-p38, and anti-phospho-p38, and HRP-conjugated anti-mouse IgG secondary Ab for β-actin at room temperature for 1 h. After incubation, the membrane was washed thrice with PBS-T, and the immunoreactive proteins were visualized with an electrochemical luminescence (ECL) detection system (EZ-Western Lumi La; Dogen, Seoul, Republic of Korea). The bands were analyzed for density using Image J software v.1.5 (NIH, Bethesda, MD, USA). Results were presented as relative intensity and each band was adjusted to that of β-actin.

### 4.7. Statistical Analysis

Data are presented as mean ± SD. An unpaired two-tailed t-test was used to compare the two groups. Statistical analysis was carried out using GraphPad InStat version 5.01 (GraphPad Software, La Jolla, CA, USA). *p* values < 0.05 were considered statistically significant.

## 5. Conclusions

Overall, this study confirms that IL-32 is induced by ENO1 stimulation in Con A-activated PBMCs and RA PBMCs. The NF-κB and p38 MAPK pathways were activated by ENO1 stimulation, and IL-32 expression and production were inhibited in PBMCs treated with NF-κB and p38 MAPK inhibitors. Thus, it can be concluded that ENO1 plays an important role in inflammation through the induction of IL-32 production by the activation of the NF-κB and p38 MAPK pathways.

## Data Availability

Data are contained within the article.
